# Management of a rare entity; a intramuscular caecal epidermoid cyst, in the robotic surgery era: a video case report

**DOI:** 10.1007/s00384-026-05104-y

**Published:** 2026-02-07

**Authors:** Taya Keating, Katie Giblin, Olubunmi Ipadeola, Carolyn Cullinane, Hazim Eltyeb, Christina Fleming

**Affiliations:** 1 Royal College of Surgeons, Ireland, RCSI, Dublin, Ireland; 2 Department of Colorectal Surgery, University Hospital Limerick, Limerick, Ireland; 3 Department of Histopathology, University Hospital Limerick, Limerick, Ireland; 4 ESCP Robotic Colorectal Surgery Fellowship Programme, University Hospital Limerick, Limerick, Ireland; 5 School of Medicine, University of Limerick, Limerick, Ireland

**Keywords:** Robotic surgery, Minimally invasive, Epidermoid cyst, Caecum, Case report

## Abstract

This case “Management of an intramuscular caecal epidermoid cyst in the robotic surgery era” documents the work-up and management of a rare entity, with the aid of the robotic surgery platform. There are 12 reported cases in the literature of epidermoid cysts arising from the caecum since 1961. Ranging from 8- to 75-year-olds, four of the epidermoid cysts were intramuscular, with six being subserosal. Given the rarity of this entity and the pre-operative diagnostic uncertainty, often both CT and MRI are used during pre-operative work-up. To our knowledge, this is the first reported case of intramuscular caecal epidermoid cyst excised using a robotic surgery approach.

**Supplementary Information:**

The online version contains supplementary material available at 10.1007/s00384-026-05104-y.

## Introduction: patient information and clinical findings

A 35-year-old female was referred for gynaecological assessment of dysmenorrhea and pelvic pain. Her past surgical history was relevant for open appendicectomy and caesarean section. She had no other past medical history. She underwent a pelvic ultrasound which identified an 8.2-cm solid pelvic mass. Further assessment was recommended with MRI pelvis.

## Timeline and diagnostic assessment

MRI pelvis demonstrated a heterogenous T2 hyperintense mass which appeared irregularly walled with a small peripheral low signal rim. It lay immediately anterior to the uterus and superior to the bladder. And there was no definite connection identified to the adjacent uterus (see Image 1).

CT abdomen and pelvis demonstrated a broad plane of contact with the caecum and minor contact with adjacent small bowel loops but no evidence of invasion into adjacent structures. The tissue of origin remained unclear at this point, and a colonoscopy was performed (see Image 2).

Colonoscopy to the caecum demonstrated a normal appearing ileocecal valve and terminal ileum. In the caecum pole, the mucosa appeared normal, but there was evidence of an external mass indenting into the caecal pole. When performing a biopsy, the mucosa easily lifted from a hard lesion underlying it (see Images 3–6).

## Therapeutic intervention

Following discussion at the GI multidisciplinary meeting, it was agreed this was likely an intramuscular caecal mass; the patient underwent a robotic-assisted right hemicolectomy with intracorporeal anastomosis. The patient was positioned supine, and an initial 4-cm Pfannenstiel incision was made. A small Alexis retractor with cap was placed, and a 10-mm Airseal assistant port inserted port. The three 8-mm ports and 12-mm camera port were also used (see Image 7).

Post-operative histological analysis revealed a caecal intramuscular epidermoid cyst (see Image 8). There were no features of malignancy, and the proximal and distal resection margins were unremarkable. Epidermoid cysts are benign, encapsulated, subepidermal nodules filled with keratin material. Although most commonly located on the face, neck, and trunk, epidermoid cysts can be found anywhere in the body.

## Follow-up and outcome

The patient followed our institution’s Enhanced Recovery After Surgery protocol and was discharged home on day four without any post-op complications. At outpatient review 4 weeks post-op, she was clinically well with healed wounds and routine bloods were normal. She will continue on B12 monitoring and replacement as required with her GP.

## Discussion

There are 12 reported cases in the literature of epidermoid cysts arising from the caecum since 1961. Ranging from neonates to 75-year-olds, the epidermoid cysts were found to be intramuscular or subserosal. Interestingly, three of the patients had had previous abdominal surgery, as did this patient, and these cases were attributed to iatrogenic implantation of epidermal fragments of tissue intra-op. While the remaining nine patients were considered to have congenital epidermoid cyst lesions. Four cases were excised using the laparoscopic approach [1–4]. To our knowledge, this is the first reported case of intramuscular caecal epidermoid cyst excised using a robotic surgery approach.

Four reports exist surrounding malignant transformation from epidermoid cysts; the patients were diagnosed with squamous cell carcinoma, highlighting the need for complete removal of the tumour [5–8]. Given the rarity of this entity and the pre-operative diagnostic uncertainty, often both CT and MRI are used during pre-operative work-up.

## Patient perspective

“Before surgery, my symptoms would flare up mainly around the time of my period and had a huge impact on my quality of life. The pain was horrific. I was essentially bedbound for the first two days of each cycle and needed prescribed pain and anti-inflammatory medication just to cope. During those days, I couldn’t work or care for my children. I also had strong ‘IBS-like’ symptoms that added to the discomfort. At first, my GP suggested trying birth control, assuming the cause might be endometriosis. But I felt uneasy about only managing the symptoms rather than addressing the root cause. When a mass was eventually found, I was quite worried, especially given the size and since none of the scans could clearly identify what it was. Dr. Fleming was very reassuring and helped ease my anxiety, though I was still nervous about the small risk of needing a stoma bag, particularly as a family member had recently gone through that experience.

The surgery itself and my recovery went really smoothly. Having had an emergency C-section before, I actually found this recovery much easier. The first day was tough. I felt very nauseous, lightheaded, and even struggled to sip water or chew gum but things improved quickly after that. Having a private hospital room made a big difference, as it allowed me to get proper rest and sleep. I also think being active and doing regular strength training beforehand really helped with how quickly I recovered. Since the surgery, my pain and IBS-like symptoms have completely disappeared. I’ve gone from being stuck in bed for two days every month to being able to go to the gym on day one of my period. It’s been life-changing and has had a huge positive impact on my wellbeing.”

## Supplementary Information

Below is the link to the electronic supplementary material.

ESM 1 (141 MB MOV)

ESM 2 (0.99 MB PNG)

ESM 3 (580 KB PNG)

ESM 4 (2.08 MB PNG)

ESM 5 (116 KB PNG)

ESM 6 (1.59 MB PNG)

## Data Availability

Data is available upon reasonable request, without patient identifiable details to maintain GDPR.
