# Supplementary figures and images for: Management of a rare entity; a intramuscular caecal epidermoid cyst, in the robotic surgery era: a video case report
Source: Int J Colorectal Dis. 2026 Feb 7;41(1):62. doi: 10.1007/s00384-026-05104-y (PMC12886303; doi:10.1007/s00384-026-05104-y)

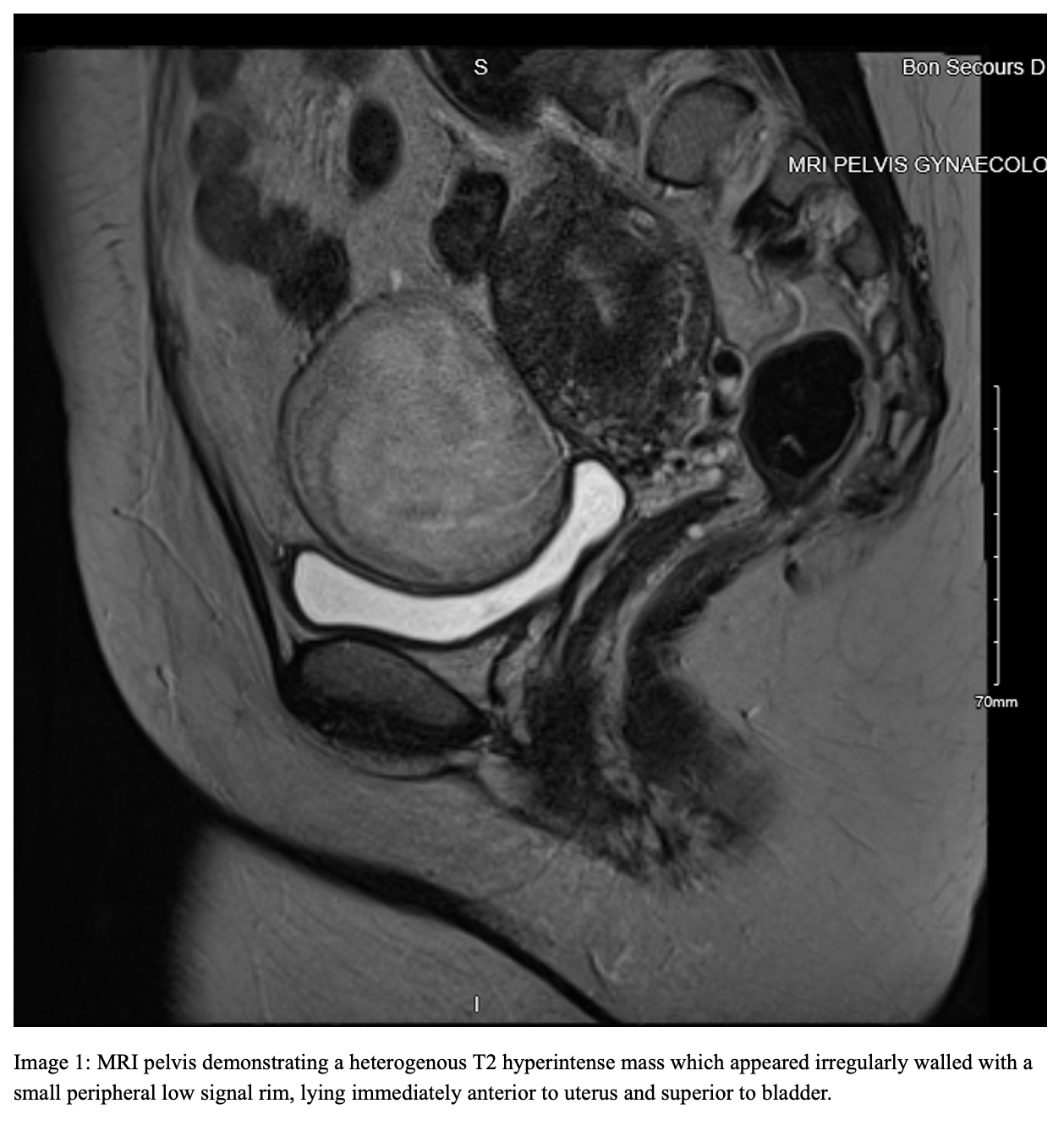

Supplement: Supplementary file 2 — (0.99 MB PNG) [file 384_2026_5104_MOESM2_ESM.png]

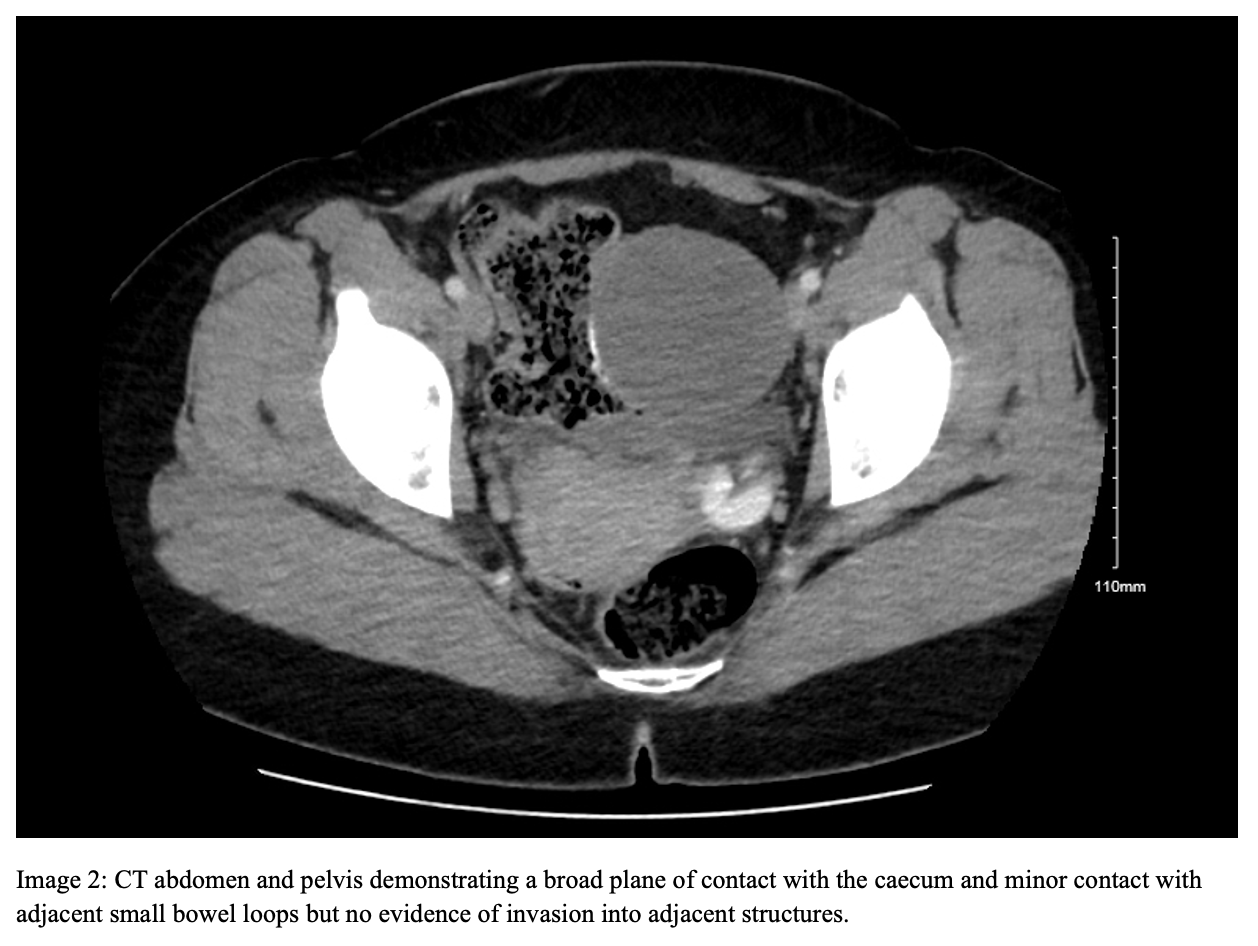

Supplement: Supplementary file 3 — (580 KB PNG) [file 384_2026_5104_MOESM3_ESM.png]

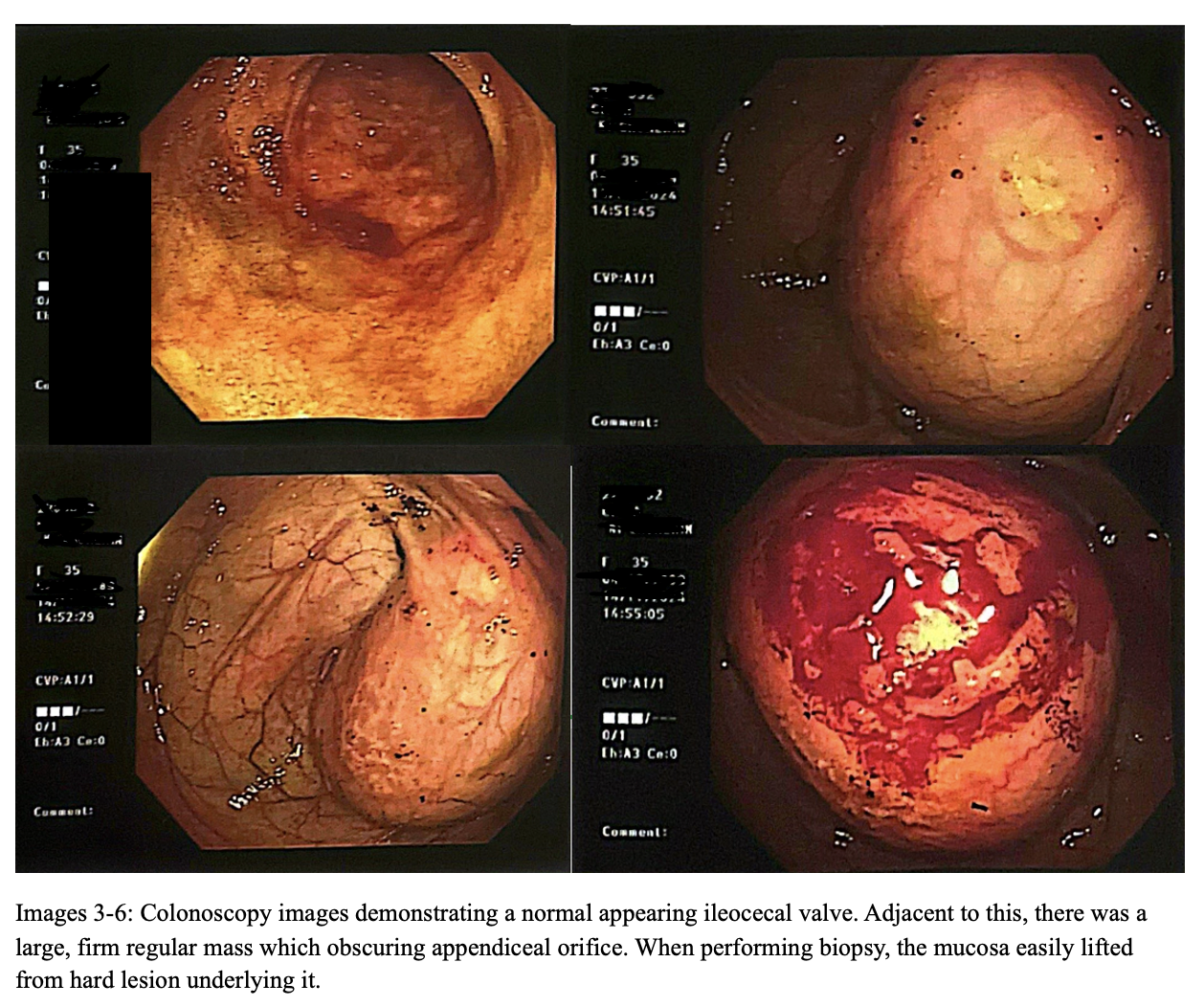

Supplement: Supplementary file 4 — (2.08 MB PNG) [file 384_2026_5104_MOESM4_ESM.png]

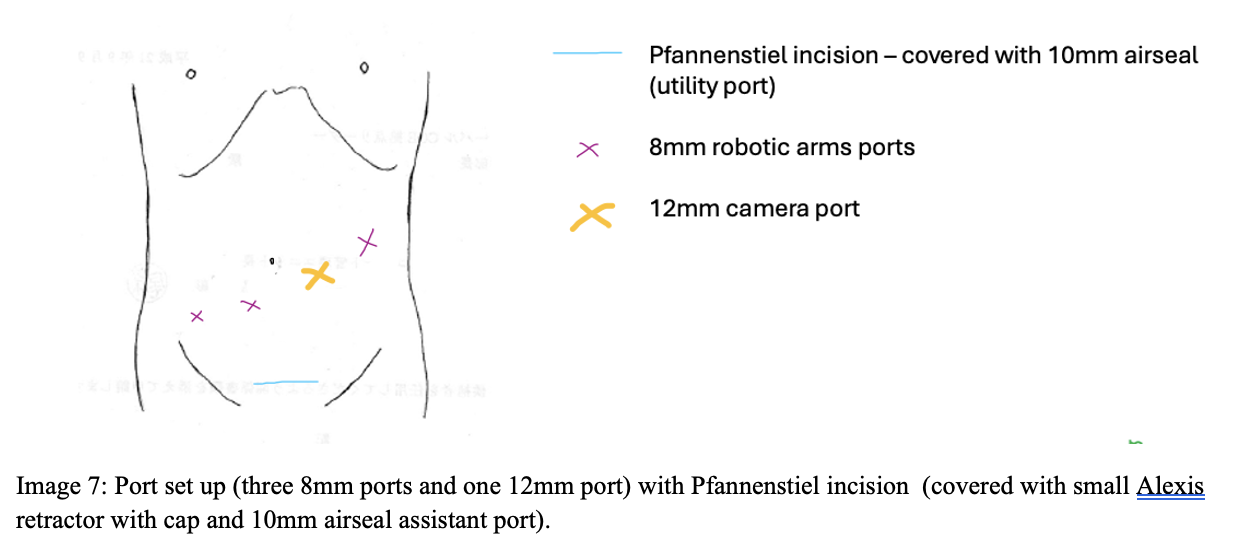

Supplement: Supplementary file 5 — (116 KB PNG) [file 384_2026_5104_MOESM5_ESM.png]

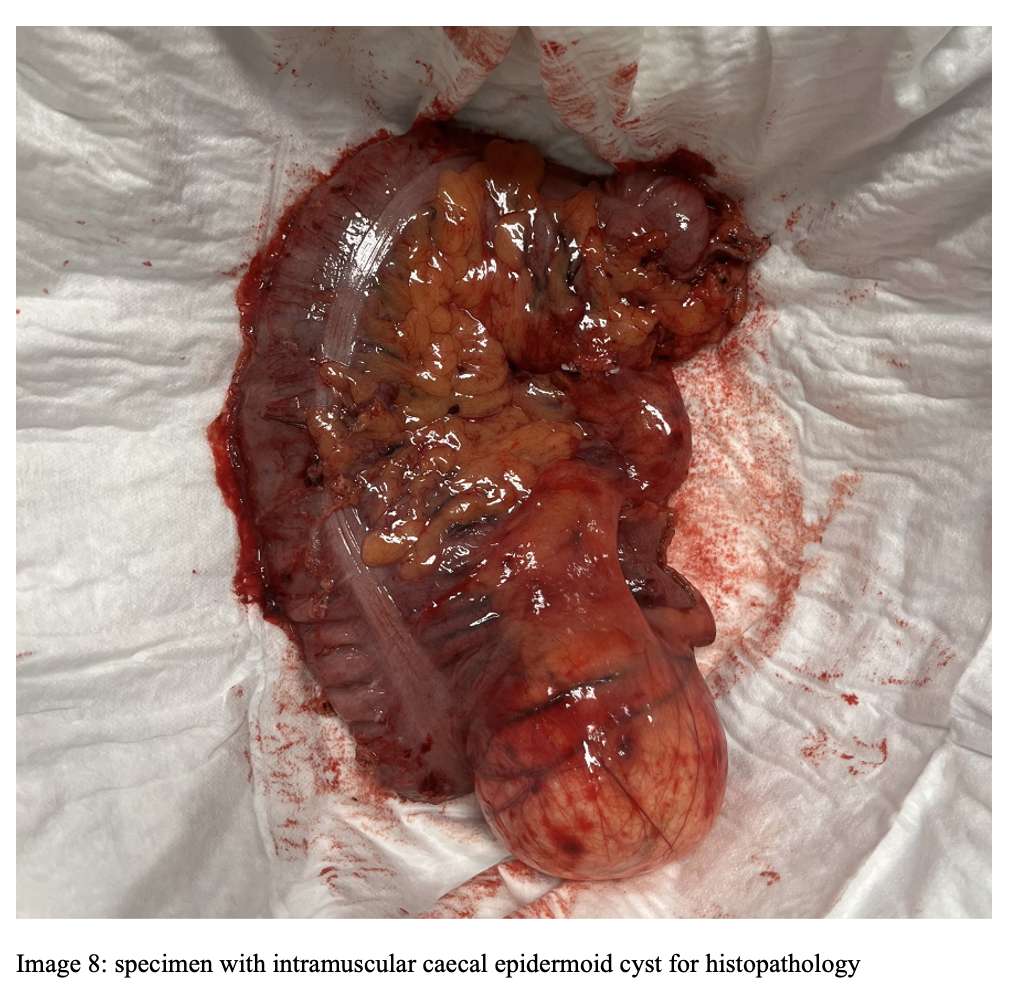

Supplement: Supplementary file 6 — (1.59 MB PNG) [file 384_2026_5104_MOESM6_ESM.png]
